# Longitudinal changes in serum adropin levels and liver fat content during liraglutide treatment in newly diagnosed patients with type 2 diabetes mellitus and metabolic dysfunction-associated fatty liver disease

**DOI:** 10.1007/s00592-023-02082-3

**Published:** 2023-04-20

**Authors:** Lin Zhang, Xiaojuan Wu, Xinyue Li, Xiaona Chang, Xiaoyu Ding, Qiu Wang, Tao Jiang, Guang Wang, Jia Liu

**Affiliations:** 1 Department of Endocrinology, Beijing Chaoyang Hospital, Capital Medical University, Beijing, 100020 China; 2 Department of Radiology, Beijing Chaoyang Hospital, Capital Medical University, Beijing, 100020 China

**Keywords:** Adropin, Liver fat content, Liraglutide, Metabolic dysfunction-associated fatty liver disease, Type 2 diabetes mellitus

## Abstract

**Aims:**

To explore the effect of liraglutide treatment on serum adropin and its relationship to the liver fat content in newly diagnosed patients with type 2 diabetes mellitus (T2DM) and metabolic dysfunction-associated fatty liver disease (MAFLD).

**Methods:**

Serum adropin level and liver fat content were assessed in patients with T2DM and MAFLD (n = 22), along with healthy controls (n = 22). Afterward, the patients received liraglutide treatment for 12 weeks. Serum adropin levels were examined by a competitive enzyme-linked immunosorbent assay. Liver fat content was quantified via magnetic resonance imaging-estimated proton density fat fraction (MRI-PDFF).

**Results:**

We found that patients with newly diagnosed T2DM and MAFLD had lower serum adropin levels [2.79 ± 0.47 vs. 3.27 ± 0.79 ng/mL, *P* < 0.05] and higher liver fat content [19.12 ± 9.46 vs. 4.67 ± 0.61%, *P* < 0.001], compared to healthy controls.

Following 12-week liraglutide treatment, serum adropin levels increased from 2.83(2.44, 3.24) to 3.65(3.20, 3.85) ng/mL (*P* < 0.001), and liver fat content decreased from 18.04(11.08, 27.65) to 7.74(6.42, 13.49) % (*P* < 0.001) in patients with T2DM and MAFLD. Furthermore, increases in serum adropin were strongly associated with decreases in liver fat content (β = − 5.933, *P* < 0.001), liver enzyme and glucolipid metabolism parameters.

**Conclusion:**

The increase in serum adropin level following liraglutide treatment was strongly correlated with the reduction in liver fat content and glucolipid metabolism. Hence, adropin might be a potential marker for the beneficial effects of liraglutide on treating T2DM and MAFLD.

## Introduction

Metabolic dysfunction-associated fatty liver disease (MAFLD), formerly known as non-alcoholic fatty liver disease (NAFLD), has become a leading cause of chronic liver disease in the world [1–3]. MAFLD frequently coexists with type 2 diabetes (T2DM), which can drive adverse outcomes including diabetic macro- and micro-vascular complications, liver fibrosis, cirrhosis, and hepatic carcinoma [4]. At present, there has no approved pharmacotherapy for MAFLD. Liraglutide, a long-acting analog of glucagon-like peptide-1 (GLP-1), is effective in treating T2DM. Recent animal studies indicate that liraglutide reduces liver fat [5], hepatic steatosis [6], inflammation [7], and oxidative stress [8] in animal models of NAFLD. Clinical evidence shows that liraglutide ameliorates body weight [9], liver fat content (LFC) [10, 11], hepatic enzymes and improves liver histology [12] in patients with NAFLD. Hence, liraglutide is expected to be a promising agent of NAFLD, but the underlying mechanisms is still unknown.

Adropin, encoded by the energy homeostasis-associated (Enho) gene, is highly expressed in the liver and brain [13]. Accumulating evidence has illustrated that adropin has been linked to metabolism and energy homeostasis. Epidemiological studies have demonstrated that circulating adropin decreased in T2DM, coronary artery disease, polycystic ovary disease, and hypertension [14–17]. Our previous study has shown that serum adropin concentration reduced and inversely correlated with NAFLD activity score (NAS) in MAFLD patients with T2DM [18]. Kumar et al. found that adropin treatment alleviated hepatic steatosis and insulin resistance, reduced expression of hepatic lipogenic genes, and improved glucose homeostasis in diet-induced obese (DIO) mice [19]. These studies collectively show that adropin might be a potential biomarker for the pathophysiology of obesity-related metabolic diseases.

This study was designed to explore the effect of liraglutide treatment on serum adropin and its relationship to the liver fat content in newly diagnosed patients with T2DM and MAFLD. Given the invasiveness, high cost and complications of liver biopsy, we conducted magnetic resonance imaging-estimated proton density fat fraction (MRI-PDFF), an emerging surrogate to diagnose and monitor the treatment response of NAFLD, to qualify liver fat content of all participants.

## Methods

### Participants

This 12-week prospective study was performed in the Department of Endocrinology in Beijing Chao Yang Hospital of Capital Medical University during the period from October 2018 to November 2019. We consecutively recruited 22 newly diagnosed patients with T2DM complicated by MAFLD, along with 22 age-, sex-matched healthy controls. Diagnosis of T2DM is based on the American Diabetes Association standard [20]. Patient selection met the following criteria: (1) diagnosed with T2DM within the previous 3 months and not use medications to treat diabetes; (2) age: 20–65 years; (3) body mass index (BMI) ≥ 24 kg/m^2^ [21]; (4) Hemoglobin A1c (HbA_1c_) ≥ 6.5% (48 mmol/mol); (5) liver fat content > 5.5% [22]. Participants were excluded for alcoholic hepatitis, drug-induced liver disease, autoimmune hepatitis, hepatocellular carcinoma, acute infectious diseases, acute myocardial infarction, hematological disorders, stroke, and any magnetic resonance imaging (MRI) contraindications.

### Study design

#### Baseline

All participants underwent a clinical assessment including comprehensive medical history, anthropometric measurement, biochemical measurements, and MRI-PDFF. Eligible patients with T2DM complicated by MAFLD receive liraglutide for 12 weeks. Liraglutide was subcutaneously injected at a starting dose of 0.6 mg/d and increased by weekly to 1.8 mg/d. Meanwhile, all patients received the recommendations of appropriate management protocols by the current guidelines, including diabetes education, diet, and exercise.

#### Study visits

Patients attended regular follow-up every 4 weeks and took records of their symptoms and medication use. The adverse events were recorded all the time. During the treatment, 1(4.5%) developed mild upper gastrointestinal upset and 1(4.5%) developed diarrhea; patients improved after symptomatic treatment. After completion of 12-week liraglutide administration, clinical assessment and fasting blood samples were assessed again.

#### Ethics

Ethical approval complying with the Helsinki Declaration was given by the Ethics Committee of Beijing Chao-yang Hospital, Capital Medical University. All participants voluntarily signed written informed consent statements prior to study initiation.

### Clinical assessment

Anthropometric characteristics (height, weight) were measured by a professional. The body mass index (BMI) was calculated as BMI = kg/m^2^. Fasting blood samples were undertaken and stored at − 80 °C. Blood samples were assayed in the central laboratory of the hospital for the following indices: lipid profile[total cholesterol (TC), high-density lipoprotein cholesterol (HDL-C), low-density lipoprotein cholesterol (LDL-C), triglyceride (TG)]; fasting blood glucose (FBG); fasting insulin (FINS); HbA1c; liver function test [alanine aminotransferase (ALT), aspartate aminotransferase (AST), γ-glutamyl transferase (GGT), total bile acids (TBA)]; and free fatty acid (FFA). Homoeostasis model assessment of insulin resistance (HOMA-IR) = FPG (mmol/L) × FINS (mU/L)/22.5; Homoeostasis model assessment of β-cell function (HOMA-β) = 20 × FINS (mU/L)/FPG (mmol/L)—3.5 [23].

Serum adropin levels were quantified by a competitive human adropin enzyme-linked immunosorbent assay (ELISA) kit (Phoenix Pharmaceuticals, Burlingame, CA, USA). The kit was used according to the manufacturers’ protocols with a sensitivity of 0.3 ng/mL. The serum adropin standards ranged from 0.3 and 8.2 ng/mL. The coefficient of variation were 10% intra-assay and 15% inter-assay.

### MRI-PDFF protocols

#### MRI examination

MRI-PDFF, a quantitative, accurate, and non-invasive imaging-based biomarker, enables us to measure the liver lipid content reproducibly [24–27]. In this study, imaging assessments were performed at baseline and weeks 12 by an experienced MRI technologist. An upper-abdominal MRI scan for each participant was performed under 12 h fasting conditions using an identical equipment set-up throughout the study. All spectra were acquired on a whole body 3-Tesla MRI scanner (Siemens Medical Solutions, Erlangen, Germany). MRI scanner protocol are shown below: (1) the localizer images were set initially; (2) a T1 volumetric interpolated breath-hold examination (VIBE) Dixon sequence was adopted with parameters: echo time (TE) 1 = 1.23 ms; TE2 = 2.46 ms; repetition time (TR) = 3.97 ms; bandwidth (BW) 1 = 1040 Hz/Px; BW2 = 1040 Hz/Px; flip angle = 9°; slice thickness = 3.0 mm.

#### MRI postprocessing

The MR images were transmitted through an image processing workstation to a Siemens Syngo. and to a radiologist for analysis. Fat-fraction map images were calculated from the in-phase and fat-phase raw data. The hepatic proton density fat fraction (PDFF) was measured with regions of interest (ROIs) in fat-fraction map image using the MITK 3M3 software (downloadable at http://www.mitk.org/). Tracing the liver boundaries were determined manually, excluding portal vein, inferior vena cava, bile ducts, focal hepatic lesions, and imaging artifacts. All images and data were supervised by a blinded senior radiologist.

### Statistical analysis

The data were expressed as mean ± SD or median (IQR). Statistical analyses were conducted by SPSS 23.0 (IBM Corporation, NY, USA). The characteristics at baseline were compared using the independent sample Student’s *t*-test (normally distributed data) or Mann–Whitney U test (skewed data). Within-group comparations (pre- and post-treatment) were done with paired Student’s *t*-test or nonparametric Wilcoxon test to assess the change of clinical parameters from baseline to the end in patients with T2DM and MAFLD. The Pearson or Spearman analyses coefficients and *p*-values were calculated among serum adropin levels, liver fat content and metabolic parameters at baseline. Moreover, we adopted linear mixed-effects models to evaluate the longitudinal relationship between serum adropin levels and liver fat content during 12-week treatment, which was performed by STATA 13.0 (STATA, College Station, TX). *P* < 0.05 were considered statistically significant.

## Results

### Baseline characteristics of participants

Baseline clinical characteristics of all subjects were presented in Table 1. The distribution of age and sex was similar across the two groups. Compared to the matched controls, patients with T2DM and MAFLD had higher BMI, TC, TG, FBG, FINS, HbA1c, HOMA-IR, AST, ALT, GGT, TBA, and FFA, and lower HDL-C and HOMA-β (all *P* < 0.05). Notably, the patients in pre-treatment group exhibited lower serum adropin levels [2.79 ± 0.47 vs. 3.27 ± 0.79 ng/mL, *P* < 0.05] and higher liver fat content [19.12 ± 9.46 vs. 4.67 ± 0.61, *P* < 0.001] than the control group (Table1, Fig. 2).

**Table 1:** Baseline characteristics of the study participants

	Groups		*P* value
	Matched control (n = 22)	Type 2 diabetes mellitus (n = 22)	
Age, y	40.18 ± 10.64	38.55 ± 9.33	0.674
Sex, M/F	17/5	17/5	
BMI, kg/m^2^	22.75 (20.38, 25.93)	31.00 (29.25, 36.05)	< 0.001*
TC, mmol/L	4.81 ± 0.60	5.76 ± 1.42	0.007*
LDL-C, mmol/L	2.91 ± 0.70	3.20 ± 1.24	0.349
HDL-C, mmol/L	1.37 ± 0.36	0.97 ± 0.23	< 0.001*
TG, mmol/L	1.17 (0.74, 1.66)	2.30 (1.56, 4.13)	< 0.001*
FBG, mmol/L	4.73 (4.53, 5.12)	8.59 (7.48, 11.69)	< 0.001*
FINS, μIU/mL	5.95 (4.78, 7.83)	15.25 (12.30, 18.93)	< 0.001*
HbA1c, %	5.30 (5.10, 5.30)	9.75 (7.93, 10.90)	< 0.001*
HOMA-IR	1.25 (1.17, 1.57)	6.23 (5.18, 9.02)	< 0.001*
HOMA-β	84.55 (53.46, 123.78)	48.64 (29.76, 90.70)	< 0.001*
AST, U/L	20.00 (17.00, 29.00)	32.50 (20.50, 50.00)	0.032*
ALT, U/L	20.50 (12.75, 36.75)	39.00 (27.75, 71.00)	0.003*
GGT, U/L	17.50 (12.50, 32.75)	59.50 (30.75, 101.75)	< 0.001*
TBA, umol/L	3.35 (2.075, 4.6)	5.25 (3.60, 7.35)	0.013*
FFA, mmol/L	0.35 (0.29, 0.50)	0.65 (0.45, 0.80)	0.002*
liver fat content, %	4.67 ± 0.61	19.12 ± 9.46	< 0.001*
Adropin, ng/mL	3.27 ± 0.79	2.79 ± 0.47	0.026*

*BMI* body mass index, *TC* total cholesterol, *LDL-C* low-density lipoprotein cholesterol, *HDL-C* high-density lipoprotein cholesterol, *TG* triglyceride, *FBG* fasting blood glucose, *FINS* fasting insulin, *HbA1c* glycosylated hemoglobin, *HOMA-IR* homeostasis model assessment of insulin resistance, *HOMA-β* homeostasis model assessment of β-cell function, *AST* alanine aminotransferase, *ALT* aspartate aminotransferase, *GGT* gamma-glutamyl transpeptidase, *TBA* total bile acids, *FFA* free fatty acid

Data shown as mean ± standard deviation were compared between two groups using Student’s *t* test for independent samples;

Data shown as median (interquartile range) were compared between two groups using Mann–Whitney U-test

The bold highlighted the significant *P* values, which makes the results easier to read and understand

### Correlations of serum adropin levels and clinical parameters

In T2DM group, serum adropin levels correlated inversely with BMI, LDL-C, TG, HbA1c, TBA, FFA and liver fat content (BMI: r = − 0.328, LDL-C: r = − 0.326, TG: r = − 0.346, HbA1c: r = − 0.298, TBA: r = − 0.537, FFA: r = − 0.316, liver fat content: r = − 0.310; all *P* < 0.05), whereas correlated positively with HDL-C (HDL-C: r = 0.616; *P* < 0.01), as described in Table 2. The control groups had only one parameter, serum TBA (r = -0.572, *P* < 0.01), negatively associated with serum adropin level.

**Table 2:** Correlation analyses of serum adropin levels with biochemical parameters in baseline

	Adropin	
	*r*	*P* value
BMI	− 0.328	0.03*
TC	− 0.016	0.919
LDL-C	− 0.326	0.031*
HDL-C	0.616	0.001*
TG	− 0.346	0.021*
FBG	− 0.167	0.279
FINS	− 0.151	0.329
HbA1c	− 0.298	0.049*
HOMA-IR	− 0.206	0.179
HOMA-β	0.001	0.993
AST	− 0.165	0.283
ALT	− 0.222	0.148
GGT	− 0.082	0.597
TBA	− 0.537	0.001*
FFA	− 0.316	0.037*
liver fat content	− 0.310	0.041*

*BMI* body mass index, *TC* total cholesterol, *LDL-C* low-density lipoprotein cholesterol, *HDL-C* high-density lipoprotein cholesterol, *TG* triglyceride, *FBG* fasting blood glucose, *FINS* fasting insulin, *HbA1c* glycosylated hemoglobin, *HOMA-IR* homeostasis model assessment of insulin resistance, *HOMA-β* homeostasis model assessment of β-cell function, *AST* alanine aminotransferase, *ALT* aspartate aminotransferase, *GGT* gamma-glutamyl transpeptidase, *TBA* total bile acids, *FFA* free fatty acid

*P < 0.05

The bold highlighted the significant *P* values, which makes the results easier to read and understand

### Effect of liraglutide on serum adropin levels, liver fat content, and metabolic parameters

Table 3 exhibited clinical and biological changes in patients with T2DM and MAFLD according to 12 weeks of treatment with liraglutide. Changes between pre- and post-treatment have shown significant decreases in BMI, TC, TG, FBG, HbA1c, HOMA-IR, AST, ALT, GGT, and FFA levels, as well as considerable increase in HOMA-β level (TC, HOMA-IR: *P* < 0.05; other indices: *P* < 0.01).

**Table 3:** Pre-treatment and post-treatment clinical characteristics of T2DM patients with MAFLD treated with liraglutide

	Group		Changes after liraglutide	*P* value
	Pre-treatment (n = 22)	Post-treatment (n = 22)		
BMI, kg/m^2^	31.00 (29.25, 36.05)	29.20 (24.84, 34.85)	− 1.99 ± 1.48	< 0.001*
TC, mmol/L	5.76 ± 1.42	5.05 ± 1.25	− 0.86 ± 1.44	0.032*
LDL-C, mmol/L	3.20 ± 1.24	3.30 ± 1.15	0.02 (− 0.45, 1.09)	0.616
HDL-C, mmol/L	0.97 ± 0.23	1.02 ± 0.18	0.09 ± 0.21	0.219
TG, mmol/L	2.30 (1.56, 4.13)	1.79 (1.29, 2.60)	− 0.60 (− 1.57, 0.29)	0.005*
FBG, mmol/L	8.59 (7.48, 11.69)	5.88 (5.41, 7.03)	− 2.75 (− 7.00,− 1.32)	< 0.001*
FINS, μIU/mL	15.25 (12.30, 18.93)	15.80 (10.68, 18.85)	− 1.70 (− 6.40, 1.40)	0.592
HbA1c, %	9.71 ± 2.09	6.16 ± 0.56	− 3.80 (− 4.70,− 1.75)	< 0.001*
HOMA-IR	6.23 (5.18, 9.02)	4.22 (3.00, 5.53)	− 1.36 (− 3.77,− 0.29)	0.007*
HOMA-β	48.64 (29.76, 90.70)	104.94 (65.74, 155.47)	53.45 (− 0.79, 95.21)	0.001*
AST, U/L	32.50 (20.50, 50.00)	21.00 (16.75, 26.50)	− 15.42 ± 16.32	0.002*
ALT, U/L	39.00 (27.75, 71.00)	22.00 (17.00, 35.50)	− 20.00 (− 31.50,− 2.50)	< 0.001*
GGT, U/L	59.50 (30.75, 101.75)	39.00 (23.75, 67.00)	− 15.00 (− 43.93,− 2.00)	0.005*
TBA, umol/L	5.25 (3.60, 7.35)	4.05 (3.08, 6.23)	0.05 (− 2.00, 1.83)	0.487
FFA, mmol/L	0.62 ± 0.23	0.40 ± 0.12	− 0.25 ± 0.24	< 0.001*
liver fat content, %	18.04 (11.08, 27.65)	7.74 (6.42, 13.49)	− 7.51 (− 11.99,− 2.49)	< 0.001*
Adropin, ng/mL	2.83 (2.44, 3.24)	3.65 (3.20, 3.85)	0.89 ± 0.73	< 0.001*

*BMI* body mass index, *TC* total cholesterol, *LDL-C* low-density lipoprotein cholesterol, *HDL-C* high-density lipoprotein cholesterol, *TG* triglyceride, *FBG* fasting blood glucose, *FINS* fasting insulin, *HbA1c* glycosylated hemoglobin, *HOMA-IR* homeostasis model assessment of insulin resistance, *HOMA-β* homeostasis model assessment of β-cell function, *AST* alanine aminotransferase, *ALT* aspartate aminotransferase, *GGT* gamma-glutamyl transpeptidase, *TBA* total bile acids, *FFA* free fatty acid

Data shown as mean ± standard deviation were compared between pre- and post-treatment using paired Student’s t test;

Data shown as median (interquartile range) were compared between pre- and post-treatment using paired Wilcoxon test;

*P < 0.05

The bold highlighted the significant *P *values, which makes the results easier to read and understand

**Fig. 1:**
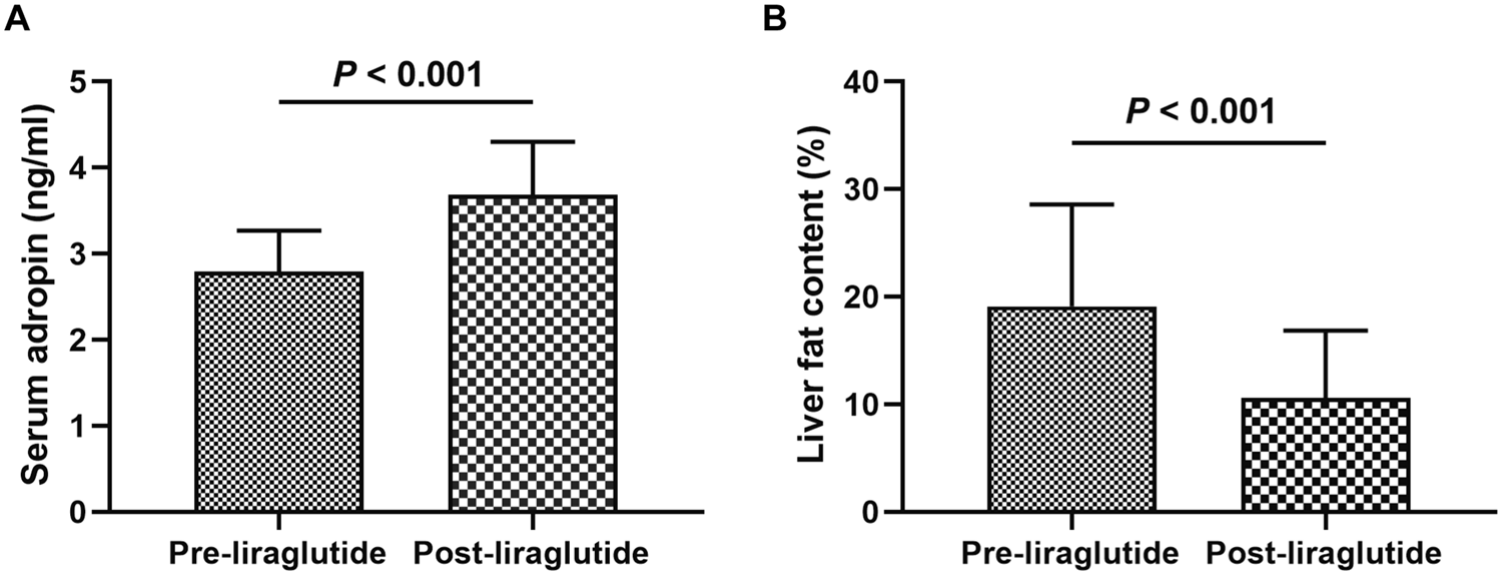
Effects of the 12-week liraglutide treatment on serum adropin levels and liver fat content. **A** Serum adropin levels; **B** liver fat content

**Fig. 2:**
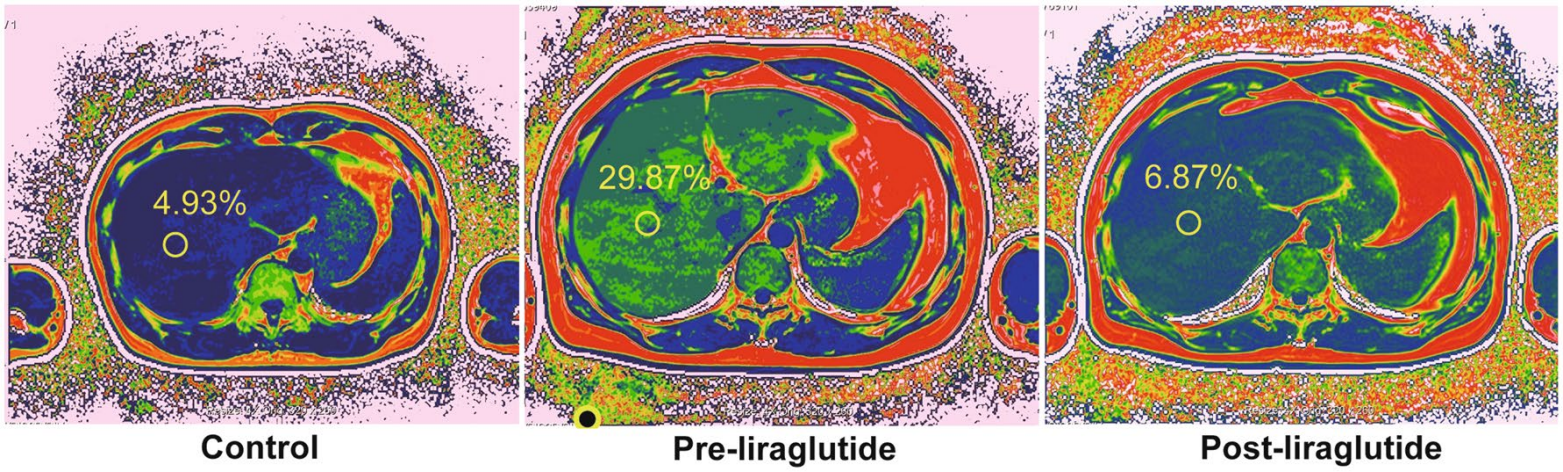
MRI-PDFF assessment among the control, pre-treatment, and post-treatment groups, respectively. The figure shows a slice from each subject and a yellow circle marked the liver fat content. The control subject is a 33 years old man who has a 4.93% liver fat content. Another sex-, age-matched patient with T2DM and MAFLD shows a decrease in PDFF from 29.87 to 6.87% following liraglutide treatment

In addition, serum adropin levels elevated from 2.83(2.44, 3.24) to 3.65(3.20, 3.85) (*P* < 0.001, Fig. 1A and Table 3) and liver fat content reduced from 18.04(11.08, 27.65) to 7.74(6.42, 13.49) (*P* < 0.001, Figs. 1B, 2 and Table 3) following liraglutide treatment.

### Correlations between elevated serum adropin and changes in liver fat content and metabolic parameters

In T2DM group, longitudinal changes by treatment were assessed by linear mixed effects models. Two-class linear mixed models were fitted, and the data were summarized in Table 4. In model 1, without adjustment, higher levels of serum adropin were related to lower BMI (β = − 2.247, *P* = 0.013), HbA1c (β = − 1.575, *P* < 0.001), FBG (β = − 1.653, *P* = 0.002), AST (β = − 7.807, *P* = 0.014), FFA (β = − 0.087, *P* = 0.047). In model 2, adjusted for sex and age, the results of BMI, HbA1c, FBG, and AST remained similar to model 1 except for FFA (β = − 0.084, *P* = 0.058) and ALT (β = − 12.611, *P* = 0.018).

**Table 4:** Associations of adropin with other parameters in mixed linear effects models

	Model 1				Model 2			
	β	LCI	UCI	*P* value	β	LCI	UCI	*P* value
BMI	− 2.247	− 4.022	− 0.472	0.013*	− 2.186	− 3.912	− 0.459	0.013*
HbA1c	− 1.575	− 2.442	− 0.708	< 0.001*	− 1.545	− 2.420	− 0.670	0.001*
FBG	− 1.653	− 2.685	− 0.622	0.002*	− 1.639	− 2.680	− 0.598	0.002*
FINS	− 1.421	− 7.373	4.532	0.640	− 1.712	− 7.475	4.051	0.560
HOMA-IR	− 1.378	0.224	− 3.599	0.844	− 1.472	− 3.639	0.695	0.183
HOMA-β	24.844	− 8.249	57.938	0.141	25.370	− 5.195	55.934	0.104
TC	− 0.455	− 1.013	0.103	0.110	− 0.478	− 1.038	0.082	0.094
HDLC	0.034	− 0.052	0.121	0.440	0.017	− 0.059	0.092	0.666
LDLC	0.037	− 0.458	0.531	0.885	− 0.009	− 0.500	0.483	0.972
TG	− 0.998	− 2.064	0.068	0.066	− 0.940	− 2.014	0.134	0.086
AST	− 7.807	− 14.038	− 1.577	0.014*	− 8.473	− 14.080	− 2.866	0.003*
ALT	− 12.042	− 24.695	0.611	0.062	− 12.611	− 23.045	− 2.177	0.018*
GGT	− 10.838	− 32.466	10.791	0.326	− 14.093	− 34.446	6.261	0.175
TBA	− 0.658	− 2.440	1.123	0.469	− 0.570	− 2.236	1.096	0.502
FFA	− 0.087	− 0.173	− 0.001	0.047*	− 0.084	− 0.171	0.003	0.058
liver fat content	− 4.938	− 8.415	− 1.460	0.005*	− 5.933	− 8.655	− 3.212	< 0.001*

*BMI* body mass index, *TC* total cholesterol, *LDL-C* low-density lipoprotein cholesterol, *HDL-C* high-density lipoprotein cholesterol, *TG* triglyceride, *FBG* fasting blood glucose, *FINS* fasting insulin, *HbA1c* glycosylated hemoglobin, *HOMA-IR* homeostasis model assessment of insulin resistance, *HOMA-β* homeostasis model assessment of β-cell function, *AST* alanine aminotransferase, *ALT* aspartate aminotransferase, *GGT* gamma-glutamyl transpeptidase, *TBA* total bile acids, *FFA* free fatty acid. *P < 0.05

Model 1 without adjustment, Model 2 adjusted for sex and age

The bold highlighted the significant *P* values, which makes the results easier to read and understand

Moreover, we observed that increases in serum adropin were strongly associated with decreases in liver fat content during 12-week liraglutide treatment even after adjusting for sex and age (β = − 5.933, *P* < 0.001).

## Discussion

In the present study, we confirmed that patients with T2DM and MAFLD had lower serum adropin levels and higher liver fat content than healthy controls. Liraglutide significantly elevated serum adropin levels, reduced liver fat content, and improved liver enzyme and other metabolic parameters. Notably, we provide the first evidence that the increased serum adropin level was strongly correlated with the decreased liver fat content following liraglutide treatment in patients with T2DM complicated by MAFLD.

Adropin, a secreted peptide, was first identified in the liver of obese mouse models in 2008 [19]. Thereafter, numerous studies have reported that adropin may be a potential regulator governing energy and metabolism homeostasis. Animal studies have shown that lower plasma adropin concentrations were observed in high-fat diet mice [28] or high-fructose diet rhesus macaques [29]. Similarly, an inverse correlation between circulating adropin concentrations and BMI was confirmed by human studies [17, 30], indicating that a low level of adropin is a hallmark of obesity.

Consistent with prior studies [17, 30, 31], our findings also suggested that serum adropin was negatively associated with BMI, LDL-C, TG, HbA1c and FFA while positively associated with HDL-C in patients with T2DM and MAFLD. Chen et al. reported that adropin-deficiency mice exhibited severe glucose homeostasis impairment and worse metabolism disorder [32]. Furthermore, the administration of synthetic adropin promotes glycogen synthesis, attenuates glucose production, and improves insulin sensitivity by raising IRS1/2-Akt phosphorylation and lowering the FoxO1 transcript in mouse models of diet-induced obesity [33]. Jasaszwili et al. [34] reported that adropin impaired preadipocyte differentiation, reduced the fat volume of brown adipose tissue, and improved the outflow of glycerol and FFA, which indicated that adropin involved in the modulation of lipid metabolism. Interestingly, we also found low serum adropin were related to high serum total bile acids, which provides a novel perspective on the role of adropin. Taken together, these results showed that low levels of adropin, which is correlated with impaired glucolipid metabolism and exacerbated insulin resistance, might be a pathogenetic factor involved in T2DM.

Multiple studies have proven that high levels of circulating adropin contributed to improved glucose tolerance, reduced insulin resistance, and ameliorated hyperlipidemia [28, 35, 36]. However, due to the high costs and time-consuming of developing new drugs, it is more effective to explore the potential of current drugs. In obese Wistar rats with T2DM, exogenous injection of adropin resulted in reduced blood glucose level, improved insulin sensitivity, ameliorated hyperlipidemia, and inhibited levels of inflammatory cytokines [37]. A recent human study of 15 obese male T2DM patients demonstrated that plasma adropin concentration increased significantly after treatment with liraglutide and metformin for three months [38]. Similarly, our results confirmed that liraglutide elevated serum levels of adropin. Simultaneously, the increased adropin was associated with weight loss, improved glucolipid metabolism, and alleviation of insulin resistance. Collectively, the results above indicated that adropin upregulation might be a novel mechanism for the beneficial effects of liraglutide in patients with T2DM.

Of note, in the current study, liver fat content was qualified by MRI-PDFF, which is considered to be a novel biomarker of MAFLD [26, 37]. In accordance with other studies [10, 12], our results demonstrated that liraglutide treatment for 12 weeks reduced liver fat content and liver enzyme, which indicated the beneficial effects of liraglutide in MAFLD. There are several possible mechanisms. First, Wu et al. [39] reported that liraglutide could ameliorate hepatic lipid accumulation via promoting reversal of cholesterol transport in diet-induced obese db/db mice. Second, Liraglutide activated autophagic flux and attenuated hepatic steatosis through the TFEB-mediated autophagy-lysosomal pathway [40]. Finally, HFD-fed genetically engineered mouse model demonstrated that liraglutide ameliorated lipid-induced hepatic steatosis though the HIF-2α/PPARα pathway [41]. Conversely, an earlier small study did not indicate an alleviation of LFC following 12-week liraglutide treatment [11]. The discrepancy might be due to differences in participants, disease duration, ongoing treatments and diagnostic techniques.

Interestingly, an inverse correlation between serum adropin levels and liver fat content was observed in patients with T2DM and MAFLD before and after liraglutide treatment. Chen et al. reported that serum adropin reduced in the non-alcoholic steatohepatitis mice and liver damage (such as hepatic steatosis and fibrosis) exacerbated in the adropin deficient mice fed with either western diet or methionine-choline deficient diet [42]. In fact, clinical studies revealed significantly low circulating adropin levels in patients with NAFLD, which indicated that adropin may involve in the pathophysiology of NAFLD [43, 44]. Moreover, animal studies revealed that adropin decreased the expression of hepatic lipogenic genes and adipose tissue *PPAR*γ gene in DIO mouse model [19, 33]. Adropin also resists oxidative stress by upregulating Nrf2, and attenuating liver injury of NASH mice [42]. In our study, the longitudinal analysis showed that an elevation of serum adropin levels was related to the decline of liver fat content and hepatic enzymes during liraglutide treatment. This result may suggest that liraglutide increased serum adropin levels, exerting significant effects on improving liver fat deposition. Taken together, these results demonstrate that adropin might be involved in the beneficial effect of liraglutide on liver steatosis in patients with MAFLD.

Our study has some limitations. First, liver fat content was quantified via MRI-PDFF rather than the gold standard liver biopsy. Given the exploratory nature of our study, performing liver biopsies even in a research setting of T2DM and MAFLD comorbidity would hardly accepted by patients who are newly diagnosed. Second, owing to the small sample size and non-randomization design, bias may have been presented; hence, larger randomized trials are warranted to validate our findings. Furthermore, further animal and cell-based experiments are required to explore the underlying molecular mechanism of liraglutide and other GLP-1 receptor agonists (dulaglutide, semaglutide) on adropin and liver fat content. Nonetheless, our findings reflected the real-world clinical settings and exhibited adequate reliability with sophisticated statistical methods.

In conclusion, our study indicated that liraglutide significantly elevated serum adropin levels in patients with T2DM and MAFLD. The increase in serum adropin level following liraglutide treatment was strongly correlated with the improvement of liver fat content and glucolipid metabolism. Hence, adropin might be a potential marker involved in the beneficial effects of liraglutide on treating T2DM and MAFLD.
